# Supplementary material for: Development of a computer-aided design software for the quantitative evaluation of aesthetic damage
Source: PLoS One. 2019 Dec 18;14(12):e0226322. doi: 10.1371/journal.pone.0226322 (PMC6919621; doi:10.1371/journal.pone.0226322)
Supplement: S1 Code site — 1. (ZIP) [file pone.0226322.s001.zip › [EESC_jr][Projeto]Relatorio_07_21/vendors/select2/docs/_includes/options/data/select.html]

## Can Select2 be used with a `<select>` tag?

Select2 was designed to be a replacement for the standard `<select>` boxes that are displayed by the browser, so by default it supports all options and operations that are available in a standard select box, but with added flexibility. There is no special configuration required to make Select2 work with a `<select>` tag.

### Does Select2 support nesting options?

A standard `<select>` box can display nested options by wrapping them with in an `<optgroup>` tag.

{% highlight html linenos %}

Nested option
{% endhighlight %}

### How many levels of nesting can there be?

Only a single level of nesting is allowed per the HTML specification. If you nest an `<optgroup>` within another `<optgroup>`, Select2 will not be able to detect the extra level of nesting and errors may be triggered as a result.

### Can `<optgroup>` tags be made selectable?

No. This is a limitation of the HTML specification and is not a limitation that Select2 can overcome. You can emulate grouping by using an `<option>` instead of an `<optgroup>` and changing the style by using CSS, but this is not recommended as it is not fully accessible.

### How are `<option>` and `<optgroup>` tags serialized into data objects?

Select2 will convert the `<option>` tag into a data object based on the following rules.

{% highlight js linenos %}
{
"id": "value attribute" || "option text",
"text": "label attribute" || "option text",
"element": HTMLOptionElement
}
{% endhighlight %}

And `<optgroup>` tags will be converted into data objects using the following rules

{% highlight js linenos %}
{
"text": "label attribute",
"children": [ option data object, ... ],
"elment": HTMLOptGroupElement
}
{% endhighlight %}
